# HPV 16 E6/E7 Promote the Glucose Uptake of GLUT1 in Lung Cancer Through Downregulation of TXNIP Due to Inhibition of PTEN Phosphorylation

**DOI:** 10.3389/fonc.2020.559543

**Published:** 2020-11-12

**Authors:** Jia-Yi Tang, Dong-Yu Li, Ling He, Xue-Shan Qiu, En-Hua Wang, Guang-Ping Wu

**Affiliations:** ^1^Department of Pathology, The First Affiliated Hospital and College of Basic Medical Sciences, China Medical University, Shenyang, China; ^2^Key Laboratory of Pathogenesis, Prevention and Therapeutics of Aortic Aneurysms, Department of Vascular and Thyroid Surgery, The First Affiliated Hospital of China Medical University, Shenyang, China

**Keywords:** human papillomavirus, glucose transporter 1, lung cancer, glucose uptake, thioredoxin interacting protein

## Abstract

High-risk human papillomavirus (HPV) infection play an important role in the development of lung cancer. Our previously study showed that E6 and E7 in HPV16 upregulated the expression of GLUT1 in lung cancer cells. However, whether they can promote the glucose uptake by GLUT1 and the underlying molecular mechanism has not been identified. It has been reported that thioredoxin interacting protein (TXNIP) regulates both the expression of GLUT1 and its glucose uptake. We speculate that high risk HPV16 infection may be closely related to TXNIP expression. Therefore, we associate HPV16 with TXNIP to explore the potential molecular mechanism of their regulation of GLUT1 expression and glucose uptake. Using double directional genetic manipulation in lung cancer cells, we showed that HPV16 E6/E7 proteins downregulated the expression of p-PTEN in lung cancer cells, the knockdown of PTEN further inhibited the expression of TXNIP, the inhibition of TXNIP further promoted the accumulation of HIF-1α by inhibiting the translocation of nuclear HIF-1α to the cytoplasm, and subsequently upregulated the expression of GLUT1 at the protein and mRNA levels. More interestingly, we found that the knockdown of TXNIP played a decisive role to promote the glucose uptake by GLUT1. Together, these findings suggested that the PTEN-TXNIP-HIF-1α axis might be related to the E6/E7-mediated expression of GLUT1 and its glucose uptake.

## Introduction

In 1980, Syrjänen first proposed the hypothesis of the role of HPV infection in the occurrence of bronchosquamous cell carcinoma (1). With the rapid development of molecular biology technology and the deepening of lung cancer research, researchers found that HPV16 is the main type of infection, in which E6 and E7 proteins are the main oncogenes, and only long-term persistent infection is closely related to the occurrence of lung cancer (2–5). Although it has not been confirmed that HPV infection is related to the occurrence of lung cancer in the western population so far (6), a considerable number of infection rates (37.22%) in Asia, especially in China, are closely related to the occurrence of lung cancer (7). Lee et al., showed that oncogenic HPV16 E6 inhibited the phosphorylation of PTEN at S380, which resulting in loss of the activity of PTEN in cervical cancer (8). However, the molecular mechanisms that regulate phosphorylation of PTEN at S380—in response to the high-risk HPV16 E6/E7 oncogene—in lung cancer, are remain unclear.

Noted, phosphatase and tensin homolog (PTEN) has been identified as a tumor suppressor gene that is mutated in a large number of cancers at high frequency. Unlike most protein tyrosine phosphatases, PTEN is known to preferentially dephosphorylate phosphoinositide substrates. Activated PTEN has been reported to be function as a tumor suppressor which was negatively regulated in the Akt/PKB signaling pathway. Shen et al., had suggested that PTEN might indirectly upregulate TXNIP at the protein level by inhibiting the phosphorylation of Akt, which triggered the activation of TXNIP in liver cancer (9). However, the PTEN-mediated regulation of TXNIP in lung cancer remains unknown.

Thioredoxin interacting protein (TXNIP), also known as vitamin D3-upregulated protein (VDUP-1) (10), negatively regulates glucose uptake of cells through a negative feedback loop (11, 12). Wu et al. revealed that TXNIP was found both in the nucleus and the plasma membrane (11). TXNIP located in the nucleus may induce the nuclear export of HIF-1α by enhancing the interaction between the von Hippel Lindau tumor suppressor (pVHL) and HIF-1α. Subsequently, HIF-1α was degraded by 26S proteasome complex in the cytoplasm (13). According to our previous findings, inhibition of HIF-1α significantly downregulated the expression of GLUT1 at both the protein and mRNA levels in lung cancer cells (14, 15). Thus we speculated that TXNIP eventually played the inhibition role in glucose uptake function of GLUT1. The TXNIP located in plasma membrane may inhibit the glucose uptake function of GLUT1 through endocytosis (11).

The aim of current study was to elucidate the regulatory mechanism between HPV16E6/E7, PTEN, TXNIP, HIF-1α, and GLUT1 in lung cancer cells and provide a new strategy for the treatment of HPV-related lung cancer. In the present study, we revealed for the first time that both E6 and E7 proteins in lung cancer cells induced the loss of PTEN activity and decreased the expression of TXNIP as well. Subsequently the downregulated TXNIP promoted the accumulation of HIF-1α in the nucleus, which further resulted in upregulated the expression of GLUT1 at protein and mRNA levels, and promoted the glucose uptake of GLUT1.

## Materials and Methods

### Cell Culture and Plasmids

H1299 and A549 cell lines used in this study were obtained from ATCC (Manassas, VA, USA). Authentication of the cell lines was verified using short tandem repeat (STR) profiling (for certification, please refer to the supplementary information). H1299 and A549 cells were cultured in HyClone (Logan, UT, USA) RPMI 1640 medium containing 10% fetal bovine serum, at 37°C in a 5% CO_2_ humidified atmosphere. Before transfections or interference, cells were cultured in a 6-well plate for 24 h.

Our previous results showed that H1299 is a low-E6- and E7-expressing cell line, whereas A549 is a high-E6- and E7-expressing cell line (13). The pEGFP-N1-HPV16 E6, pEGFP-N1-HPV16 E7, and pEGFP-N1 plasmids were gifted by Professor Xudong Tang, from the Department of Biochemistry and Molecular Biology, Guangdong Medical College, China. HPV16 E6 siRNA (Si-h-E6_001), HPV16 E7 siRNA (Si-h-E7_002), TXNIP siRNA (St-h-TXNIP_001), and PTEN siRNA (St-h-PTEN_001) were purchased from RiboBio (Guangzhou, China). Scrambled siRNA was used as a non-specific siRNA control.

### Transfection

Plasmids with the respective target sequence were transiently transfected into cells using the Lipofectamine 3000 Transfection Kit (Invitrogen, Carlsbad, CA, USA). Transfections with empty vector, as well as mock transfections were served as appropriate controls. Transfection efficiency was evaluated by directly visualizing cells expressing the green fluorescent protein (GFP) under fluorescence microscope. Protein content was assessed 48 h after transfection using western blotting. mRNA analysis was performed 24 h after transfection using quantitative real-time reverse transcription-polymerase chain reaction (qRT-PCR).

### Western-Blot Assays

The Western-blot assays described in reference with PMID 31839825^14^. HPV16 E6 (1:200, Bioss Biotechnology Co., Ltd, Beijing, China), HPV16 E7 (1:200, Bioss Biotechnology), PTEN (total protein, 1:500, Wanleibio Co., Ltd, Shenyang, China), p-PTEN-S380 (serine 380 phosphorylated protein, 1:1000, Zenbio Co., Ltd., Chengdu, China), TXNIP (1:500; Proteintech Co., Ltd, Wuhan, China), HIF-1α (1:1000; Wanleibio), GLUT1 (1:500; Wanleibio), and GAPDH (1:1000, Cell Signaling Technology, Danvers, MA, USA). Membranes were further incubated with peroxidase-coupled anti-mouse or anti-rabbit IgG (1:5000; Proteintech) at 37°C for 2 h. Bound proteins were visualized using the ECL Western blot kit (advansta, USA), and their densities were measured using a BioImaging systems (UVP Inc., Upland, CA, USA). Protein bands were visualized using electrochemiluminescence substrate (Pierce) and detected by using BioImaging Systems (DNR, Jerusalem, Israel). GAPDH protein levels were used as the control group to calculate relative protein levels.

### Quantitative Real-Time PCR Assays

The Quantitative real-time PCR assays described in reference with PMID 31839825 (14).

Detailed information of the primers is given in **Table 1**. The amplified products of E6, E7, PTEN, TXNIP, HIF-1α, GLUT1, and GAPDH were confirmed by correct sizes on an agarose DNA gel. Products were extracted and purified from the gel, and sent for DNA sequencing, respectively. The sequencing results were 100% correct.

**Table 1:** Sequences and features of primers used for qRT-PCR.

Gene	Forward/Reverse	Sequence	Size (bp)	mRNA
E6	270	GTATGGAACAACATTAGAACAGCAA	79	KX545363
	349	GTGGCTTTTGACAGTTAATACACC		
E7	482	GCATGGAGATACACCTACATTG	273	KX545363
	754	TGGTTTCTGAGAACAGATGG		
PTEN	1113	TTTGAAGACCATAACCCACCAC	134	NM_000314.8
	1246	ATTACACCAGTTCGTCCCTTTC		
TXNIP	545	GGCGGGTGTCTGTCTCTGCT	143	NM_001313972.2
	687	GGCAAGGTAAGTGTGGCGGG		
HIF-1α	1811	AGACAAAGTTCACCTGAGCC	174	NM_001530.4
	1984	GGGAGCTAACATCTCCAAGTCT		
GLUT1	1071	CTGGCATCAACGCTGTCTTC	167	NM_006516.3
	1237	GCCTATGAGGTGCAGGGTC		
GAPDH	50 120	TTCTTTTGCGTCGCCAGCCGAG CCAGGCGCCCAATACGACCAAA	71	XM_019023188.1


mRNA, messenger RNA; qRT-PCR, quantitative real-time reverse transcriptase-polymerase chain reaction.

### Analysis of HIF-1α Nuclear Export

A549 cells were interfered with siTXNIP. Nuclear isolation was performed using the Nuclear and Cytoplasmic Protein Extraction Kit (P0028, Beyotime Biotechnology, Shanghai, China) according to the manufacturer’s instructions. Extracted nuclear levels of HIF-1α were measured by western blot assays using anti-HIF-1α (1:1,000; Wanleibio). Levels of nuclear histone protein, used as an internal standard, were measured using an anti-histone antibody (Sangon Biotech).

### Glucose Uptake Assay

Cells (2–5 × 10^4^ cells/well) were seeded 1day before starting the assay. After 12 h, the regular culture medium (with 10% FBS) was removed, and cells were either transfected with siTXNIP or left untransfected in 400-µL cell culture medium supplemented with 0.5% FBS, and incubated at 37°C with 5% CO2 for 3 h. Then, 4 mL of the 2-NBDG (2-N-[(7-nitro-2,1,3-benzoxadiazol-4-yl) amino]-D-glucose) ﬂuorescent glucose analogue (BioVision, CA, USA) was added to each sample and incubated at 37°C with 5% CO_2_ for 30 min. After the incubation, cells from the plate were kept on ice and washed once with 1 mL ice-cold 1× Analysis Buffer. Fluorescent microscopy (BX-51, Olympus Corporation, Tokyo, Japan) was used for measuring the levels of 2-NBDG. The relative level of 2-NBDG was measured using the Image J software.

### Statistical Analysis

All statistical analyses were performed using SPSS for Windows 13.0 (SPSS Inc., Chicago, IL, USA). Data represented results from 3 or more experiments. Statistical significance was determined by Student’s *t*-test, and a p value of <0.05 was considered significant.

## Results

### Both E6 and E7 Downregulated the Expression of p-PTEN and TXNIP but Upregulated the Expression of HIF-1α and GLUT1

**Figure 1:**
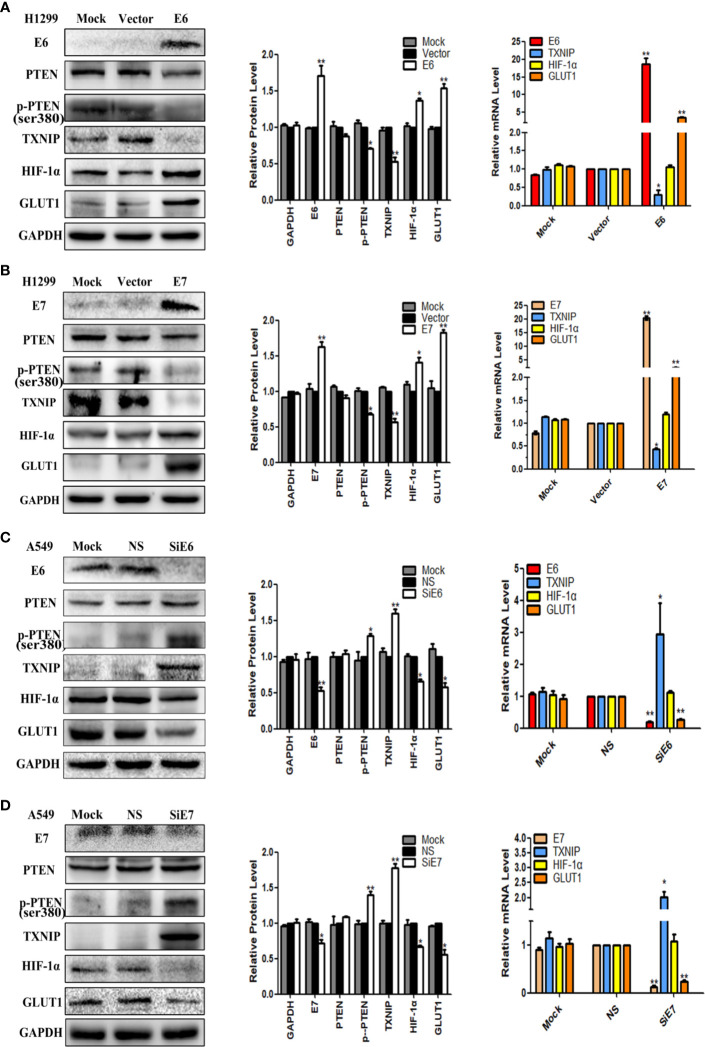
The effects of E6 and E7 on the regulation the expression levels of PTEN, p-PTEN, TXNIP, HIF-1α, and GLUT1 in lung cancer cell lines. **(A)** The expression levels of E6, PTEN, p-PTEN, TXNIP, HIF-1α, GLUT1 and GAPDH were demonstrated by western blotting and qRT-PCR in H1299 cells. **(B)** The expression levels of E7, PTEN, p-PTEN, TXNIP, HIF-1α, GLUT1 and GAPDH were demonstrated by western blotting and qRT-PCR in H1299 cells. **(C)** The expression levels of E6, PTEN, p-PTEN, TXNIP, HIF-1α, GLUT1 and GAPDH were demonstrated by western blotting and qRT-PCR in A549 cells. **(D)** The expression levels of E7, PTEN, p-PTEN, TXNIP, HIF-1α, GLUT1 and GAPDH were demonstrated by western blotting and qRT-PCR in A549 cells. Mock: mock transfection; vector: empty vector; ns: no significance (*p < 0.05; **p < 0.0l).

To investigate the role of E6 and E7, pEGFP-N1-E6 or -E7 vectors were transiently transfected into the H1299 low-expressing cell line, with the E6 or E7 empty vectors and mock transfections serving as controls. Results showed that the overexpression of E6 or E7 significantly downregulated the expression of p-PTEN and TXNIP at both protein and mRNA levels but upregulated the expression of HIF-1α at the protein level only and of GLUT1 at both the protein and mRNA levels. The expression of PTEN had little or no change. The results were presented in **Figures 1A, B**. On the other hand, the inhibition of both E6 and E7 received the opposite results in A549 cells, and they were presented in **Figures 1C, D**.

### The Knockdown of PTEN Downregulated the Expression of TXNIP but Upregulated the Expression of HIF-1α and GLUT1

**Figure 2:**
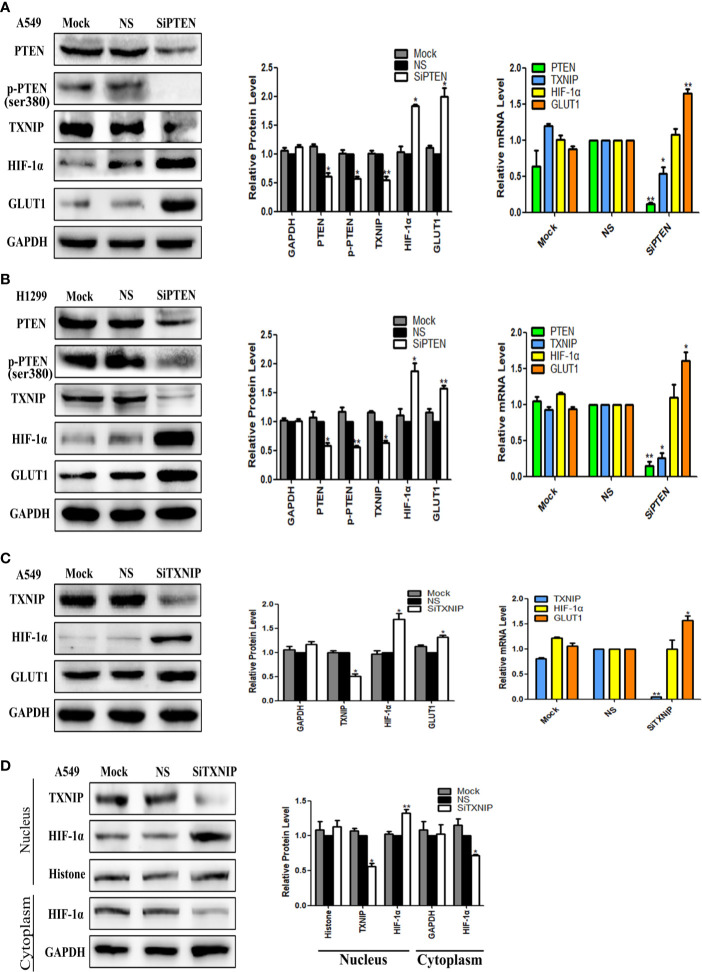
The effect of PTEN knockdown on the expression levels of TXNIP, HIF-1α, and GLUT1. TXNIP knockdown on the expression levels of HIF-1α and GLUT1, nuclear and cytoplasmic HIF-1α. The relative PTEN, p-PTEN, TXNIP, HIF-1α, GLUT1, and GAPDH levels were detected by western blotting and qRT-PCR in A549 and H1299 cells **(A, B)**. The relative expression levels of TXNIP, HIF-1α, GLUT1, and GAPDH were detected by western blotting and qRT-PCR in A549 cells **(C)**. Nuclear plasma separation technique was used to separate the proteins in nucleus and in cytoplasm. The relative levels of TXNIP, HIF-1α, GAPDH, and Histone were measured by western blotting **(D)**. Mock: mock transfection; ns: no significance (*p < 0.05; **p < 0.0l).

PTEN-specific siRNA was used to knockdown the expression of PTEN in A549 and H1299 cells. PTEN-nonspecific siRNA and mock specific siRNA served as controls. As showed in **Figures 2A, B**, use of the PTEN-specific siRNA led to downregulation the expression of p-PTEN and TXNIP at both the protein and mRNA levels, but upregulated the expression of HIF-1α at the protein level only and GLUT1 at both the protein and mRNA levels. There was no significant change observed in the PTEN-nonspecific siRNA and mock specific siRNA groups.

### The Knockdown of TXNIP Upregulated the Expression of HIF-1α and GLUT1

TXNIP-specific siRNA was used to knockdown the expression of TXNIP in the A549 cell lines, and TXNIP-nonspecific siRNA and mock specific siRNA were used as controls. The results were showed in **Figure 2C**, TXNIP-specific siRNA upregulation the expression of HIF-1α at the protein level only, whereas that of GLUT1 was elevated at both the protein and mRNA levels. There was no significant change noted in the TXNIP-nonspecific siRNA and mock specific siRNA groups.

### The Knockdown of TXNIP Upregulated the Level of HIF-1α in the Nucleus but Downregulated the Level of HIF-1α in the Cytoplasm

To further verify the regulatory role of TXNIP on HIF-1α, we knocked down the expression of TXNIP in A549 cells and separated the proteins in the nucleus from those in the cytoplasm using a nuclear plasma separation technique. The results showed that the expression level of HIF-1α was upregulated in nucleus but downregulated in cytoplasm. Results were presented in **Figure 2D**.

### The Overexpression of Both E6 and E7 and the Knockdown of Both PTEN and TXNIP Significantly Promoted the Glucose Uptake of GLUT1, Whereas the Knockdown of Both E6 and E7 Obviously Inhibited the Glucose Uptake of GLUT1

**Figure 3:**
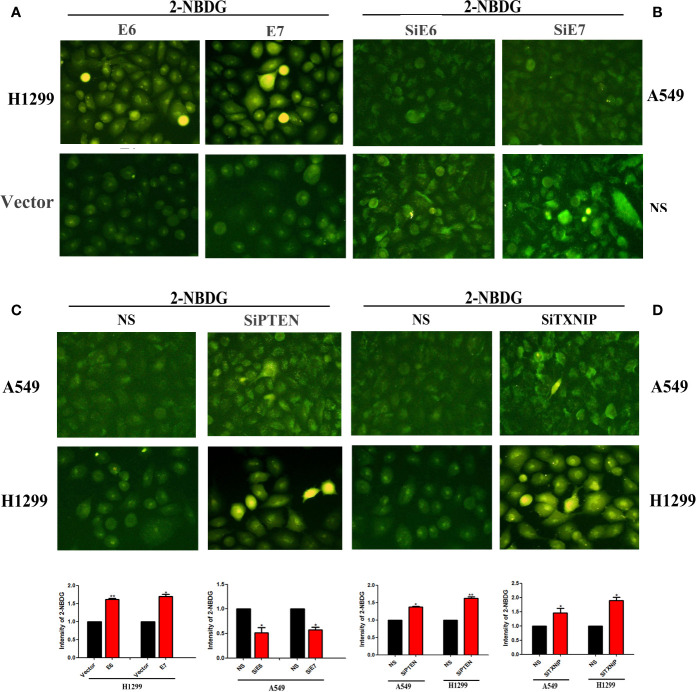
The effects of E6, E7, PTEN, and TXNIP on the amount of glucose uptake by GLUT1. Glucose uptake assay was conducted to measure the cellular uptake of glucose. The changes of glucose uptake by GLUT1 after transfection with E6 or E7 were presented by **(A)**, interference with E6 or E7 by **(B)**, interference with PTEN by **(C)**, and interference with TXNIP by **(D)**, respectively. The relative levels of 2-NBDG were measured by fluorescence microscopy and Image J software respectively. (*p < 0.05 versus NS group; **p < 0.0l versus NS group).

To investigate the functional roles of E6, E7, PTEN, and TXNIP on GLUT1, we transfected E6 or -E7 vectors in H1299 and knocked down PTEN and TXNIP in A549 and H1299 cells. Our results showed that the overexpression of both E6 and E7 and knockdown of both PTEN and TXNIP significantly promoted the glucose uptake of GLUT1 in H1299 cells. A similar but not equally intense effect was noted in the A549 cell line. More specifically, the levels of glucose uptake were significantly higher than those of the control group by fluorescence microscopy in lung cancer cells. Results were presented in **Figures 3A–D**.

## Discussion

In our previous studies, we had reported that the overexpression of HPV16 E6/E7 resulted in the upregulation of GLUT1 at protein and mRNA levels in lung cancer cells (14, 15). The mRNA expression levels of E6 and E7 in squamous cell carcinoma of the lungs were statistically higher than that in the lungs of pneumonia and tuberculosis (16). We also reported that GLUT1 promote**d** the malignant phenotype of non-small cell lung cancer through Integrin β1/Src/FAK signaling (17). A meta-analysis showed the high expression levels of GLUT1 accompanied with poor prognosis in lung cancer patients. Thus, GLUT1 may server as a biomarker and a potential target for selection of the treatment strategies of lung cancer (18). However, it is remained unclear whether E6 and E7 proteins promote the glucose uptake by GLUT1 in lung cancer cells. In this study, we provided evidences that the overexpression of E6 or E7 in lung cancer cells downregulated the expression level of p-PTEN. When knockdown of PTEN, the expression of TXNIP at both protein and mRNA levels were also significantly decreased. The inhibition of TXNIP further upregulated the protein expression of HIF-1α and promoted its accumulation in the nucleus. Consequently, HIF-1α upregulated the expression of GLUT1 at both protein and mRNA levels. The inhibition of TXNIP also promoted the glucose uptake by GLUT1 in lung cancer cells. Thus we demonstrated that TXNIP acted as a safeguard against HPV-stimulated aerobic glycolysis and tumorigenesis by inhibited its two down-stream effectors HIF-1α and GLUT1. These findings were consistent with the effects of TXNIP on HIF-1 and glucose metabolism pathways reported by Zhu et al. (19) and Sullivan et al. (20). Our results provided new molecular mechanisms in HPV16 E6/E7 promoted the glucose uptake by relieving TXNIP inhibitory effect on HIF-1α and GLUT1 in lung cancer cells.

It had been reported that the knockdown of HPV16 E6 promoted the phosphorylation of PTEN at S380 in cervical cancer (8). E6 protein through downregulation of PTEN to promote the proliferation of lung adenocarcinoma cells (21). PTEN triggered the activation of TXNIP protein by inhibiting the phosphorylation of Akt in hepatocytes (9). Our results demonstrated that the overexpression of both E6 and E7 significantly downregulated the expression of p-PTEN at S380 and the knockdown of PTEN decreased the expression of TXNIP in lung cancer cells. Wu et al. had reported that a fraction of TXNIP in the nucleus and also observed a significant fraction of TXNIP on the plasma membrane (11). The nuclear localized TXNIP was reported to mediate the nuclear export of HIF-1α and its degradation by forming a complex with HIF1α (19). Shin et al. had also shown that TXNIP promoted the nuclear export of HIF-1α by enhancing the interaction between pVHL and HIF-1α, which was subsequently degraded by the 26S proteasome complex in the cytoplasm (13). In the present study, we also found that the inhibition of TXNIP significantly upregulated the protein expression of HIF-1α in the nucleus and down regulated the protein expression of HIF-1α in the cytoplasm by nuclear cytoplasmic protein separation. Therefore, our findings confirmed that the accumulation of HIF-1α in the nucleus resulted in the upregulation of GLUT1 expression.

It is well known that cancer cells consume more glucose to gain energy through glycolysis even in aerobic conditions. This unique metabolic mode is called Warburg effect (22). GLUT1 is the main gene for cancer cells to achieve Warburg effect through glycolysis. GLUT1 is also the main transporter to transport extracellular glucose into cells. However, the expression level of GLUT1 in cytoplasm is not directly related to the activation of GLUT1. Most of the overexpressed GLUT1 are in the cytoplasm with a nonfunctional state, and they are not able to play the role of the “Porter” of glucose (23). The activation of GLUT1 is determined by two factors: one is the translocation of GLUT1 from the cytoplasm to the plasma membrane to participate in the glucose uptake (24, 25); the other is to activate the intrinsic GLUT1 on the plasma membrane to play the role of glucose uptake (26, 27). Phadngam et al showed that PTEN dephosphorylates AKT to prevent the expression of GLUT1 on plasma membrane and to limit glucose consumption in cancer cells (28). And Wu et al showed that TXNIP located in the plasma membrane mediated the inhibition of GLUT1 function through endocytosis (11). In the present study, we validated that the inhibition of TXNIP significantly promoted the glucose uptake by GLUT1 in lung cancer cells. However, it is not clear whether the knockdown of TXNIP promoted the glucose uptake by GLUT1 through HIF-1α/GLUT1 axis or through endocytosis. The detailed molecular mechanism will be further studied in the future.

In conclusion, we demonstrated that HPV16 E6/E7 proteins downregulated the expression of p-PTEN in lung cancer cells, the knockdown of PTEN further decreased the expression of TXNIP, the inhibition of TXNIP further promoted the accumulation of HIF-1α by inhibiting the translocation of nuclear HIF-1α to the cytoplasm, and consequently upregulated the expression of GLUT1 at the protein and mRNA levels. More interestingly, we confirmed that the inhibition of TXNIP played a decisive role to promote the glucose uptake by GLUT1 (29). Our findings provided new evidence in support of the critical role of TXNIP in the pathogenesis of HPV-related lung cancer, and might also suggest novel therapeutic targets.

## Data Availability Statement

The raw data supporting the conclusions of this article will be made available by the authors, without undue reservation.

## Author Contributions

J-YT, X-SQ, E-HW, and G-PW contributed to study design and conduct. J-YT, D-YL, and LH analyzed the data and provided statistical support. All authors contributed to the article and approved the submitted version.

## Funding

The present study was supported by grants from the National Natural Science Foundation of China (Grant No.81171650 and 81672082).

## Conflict of Interest

The authors declare that the research was conducted in the absence of any commercial or financial relationships that could be construed as a potential conflict of interest.

## References

[1] Syrjänen KJ Epithelial lesions suggestive of a condylomatous origin found closely associated with invasive bronchial squamous cell carcinomas. Respiration (1980) 40(3):150–60. 10.1159/000194265 10.1159/000194265 6255522

[2] Corrales L Rosell R Cardona AF Martín C Zatarain-Barrón ZL Arrieta O Lung cancer in never smokers: The role of different risk factors other than tobacco smoking. Crit Rev Oncol Hematol (2020) 148:102895. 10.1016/j.critrevonc.2020.102895 32062313

[3] Liu J Huang B Xiu Z Zhou Z Liu J Li X PI3K/Akt/HIF-1α signaling pathway mediates HPV-16 oncoprotein-induced expression of EMT-related transcription factors in non-small cell lung cancer cells. J Cancer (2018) 9(19):3456–66. 10.7150/jca.26112 PMC6171031 30310502

[4] de Oliveira THA do Amaral CM de França São Marcos B Nascimento KCG de Miranda Rios AC Quixabeira DCA Presence and activity of HPV in primary lung cancer. J Cancer Res Clin Oncol (2018) 144(12):2367–76. 10.1007/s00432-018-2748-8 PMC11813451 30225539

[5] Zhang E Feng X Liu F Zhang P Liang J Tang X Roles of PI3K/Akt and c-Jun signaling pathways in human papillomavirus type 16 oncoprotein-induced HIF-1α, VEGF, and IL-8 expression and in vitro angiogenesis in non-small cell lung cancer cells. PloS One (2014) 9(7):e103440. 10.1371/journal.pone.0103440 25058399 PMC4110025

[6] Colombara DV Manhart LE Carter JJ Hawes SE Weiss NS Hughes JP Prior human polyomavirus and papillomavirus infection and incident lung cancer: a nested case-control study. Cancer Causes Control (2015) 26(12):1835–44. 10.1007/s10552-015-0676-3 PMC4628600 26415892

[7] Yu Y Liu X Yang Y Zhao X Xue J Zhang W Effect of FHIT loss and p53 mutation on HPV-infected lung carcinoma development. Oncol Lett (2015) 10(1):392–8. 10.3892/ol.2015.3213 PMC4487131 26171037

[8] Lee EH Ji KY Kim EM Kim SM Song HW Choi HR Blockade of Axl signaling ameliorates HPV16E6-mediated tumorigenecity of cervical cancer. Sci Rep (2017) 7(1):5759. 10.1038/s41598-017-05977-8 28720772 PMC5516033

[9] Shen F Xiong Z Kong J Wang L Cheng Y Jin J Triptolide impairs thioredoxin system by suppressing Notch1-mediated PTEN/Akt/Txnip signaling in hepatocytes. Toxicol Lett (2019) 300:105–15. 10.1016/j.toxlet.2018.10.024 30394310

[10] Wang X Nachliely M Harrison JS Danilenko M Studzinski GP. Participation of vitamin D-upregulated protein 1 (TXNIP)-ASK1-JNK1 signalosome in the enhancement of AML cell death by a post-cytotoxic differentiation regimen. J Steroid Biochem Mol Biol (2019) 187:166–73. 10.1016/j.jsbmb.2018.11.015 PMC6501208 30508644

[11] Wu N Zheng B Shaywitz A Dagon Y Tower C Bellinger G AMPK-dependent degradation of TXNIP upon energy stress leads to enhanced glucose uptake via GLUT1. Mol Cell (2013) 49(6):1167–75. 10.1016/j.molcel.2013.01.035 PMC3615143 23453806

[12] Parikh H Carlsson E Chutkow WA Johansson LE Storgaard H Poulsen P TXNIP regulates peripheral glucose metabolism in humans. PloS Med (2007) 4(5):e158. 10.1371/journal.pmed.0040158 17472435 PMC1858708

[13] Shin D Jeon JH Jeong M Suh HW Kim S Kim HC VDUP1 mediates nuclear export of HIF1alpha via CRM1-dependent pathway. Biochim Biophys Acta (2008) 1783(5):838–48. 10.1016/j.bbamcr.2007.10.012 18062927

[14] Fan R Hou WJ Zhao YJ Liu SL Qiu XS Wang EH Overexpression of HPV16 E6/E7 mediated HIF-1α upregulation of GLUT1 expression in lung cancer cells. Tumour Biol (2016) 37(4):4655–63. 10.1007/s13277-015-4221-5 26508030

[15] Gu NJ Wu MZ He L Wang XB Wang S Qiu XS HPV 16 E6/E7 up-regulate the expression of both HIF-1α and GLUT1 by inhibition of RRAD and activation of NF-κB in lung cancer cells. J Cancer (2019) 10(27):6903–9. 10.7150/jca.37070 PMC6909954 31839825

[16] Zhao HY Yang JH Wang X Sun J Wang EH Wu GP Analysis of human papillomavirus 16 E6/E7 and L1 in the bronchial brushing cells of patients with squamous cell carcinoma of the lungs. Int J Clin Exp Pathol (2018) 11(8):4124–9. PMC6962774 31949804

[17] Zhao H Sun J Shao J Zou Z Qiu X Wang E Glucose Transporter 1 Promotes the Malignant Phenotype of Non-Small Cell Lung Cancer through Integrin β1/Src/FAK Signaling. J Cancer (2019) 10(20):4989–97. 10.7150/jca.30772 PMC6775508 31598171

[18] Zhang B Xie Z Li B The clinicopathologic impacts and prognostic significance of GLUT1 expression in patients with lung cancer: A meta-analysis. Gene (2019) 689:76–83. 10.1016/j.gene.2018.12.006 30552981

[19] Zhu G Zhou L Liu H Shan Y Zhang X MicroRNA-224 Promotes Pancreatic Cancer Cell Proliferation and Migration by Targeting the TXNIP-Mediated HIF1α Pathway. Cell Physiol Biochem (2018) 48(4):1735–46. 10.1159/000492309 30078003

[20] Sullivan WJ Mullen PJ Schmid EW Flores A Momcilovic M Sharpley MS Extracellular Matrix Remodeling Regulates Glucose Metabolism through TXNIP Destabilization. Cell (2018) 175(1):117–32. 10.1016/j.cell.2018.08.017 PMC6151140 30197082

[21] Mao W Li T LncRNA MACC1-AS1 Promotes Lung Adenocarcinoma Cell Proliferation by Downregulating PTEN. Cancer Biother Radiopharm (2020) 35(4):313–8. 10.1089/cbr.2019.3020 32109147

[22] Warburg O On the origin of cancer cells. Science (1956) 123(3191):309–14. 10.1126/science.123.3191.309 13298683

[23] Deng D Sun P Yan C Ke M Jiang X Xiong L Molecular basis of ligand recognition and transport by glucose transporters. Nature (2015) 526(7573):391–6. 10.1038/nature14655 26176916

[24] Roy S Leidal AM Ye J Ronen SM Debnath J Autophagy-Dependent Shuttling of TBC1D5 Controls Plasma Membrane Translocation of GLUT1 and Glucose Uptake. Mol Cell (2017) 67(1):84–95.e5. 10.1016/j.molcel.2017.05.020 28602638 PMC5522182

[25] Jing M Cheruvu VK Ismail-Beigi F Stimulation of glucose transport in response to activation of distinct AMPK signaling pathways. Am J Physiol Cell Physiol (2008) 295(5):C1071–82. 10.1152/ajpcell.00040.2008 PMC2584987 18701654

[26] Calado SM Alves LS Simão S Silva GA GLUT1 activity contributes to the impairment of PEDF secretion by the RPE. Mol Vis (2016) 22:761–70. PMC4943856 27440994

[27] Loaiza A Porras OH Barros LF Glutamate triggers rapid glucose transport stimulation in astrocytes as evidenced by real-time confocal microscopy. J Neurosci (2003) 23(19):7337–42. 10.1523/JNEUROSCI.23-19-07337.2003 PMC6740433 12917367

[28] Phadngam S Castiglioni A Ferraresi A Morani F Follo C Isidoro C PTEN dephosphorylates AKT to prevent the expression of GLUT1 on plasmamembrane and to limit glucose consumption in cancer cells. Oncotarget (2016) 7(51):84999–5020. 10.18632/oncotarget.13113 PMC5356715 27829222

[29] Hong SY Yu FX Luo Y Hagen T Oncogenic activation of the PI3K/Akt pathway promotes cellular glucose uptake by downregulating the expression of thioredoxin-interacting protein. Cell Signal (2016) 28(5):377–83. 10.1016/j.cellsig.2016.01.011 26826652

